# Novel Insights Large Estrogen and Cortisol Co-Secreting Adrenocortical Tumor in a 17-Year-Old Female: A Case Report and Literature Review

**DOI:** 10.1159/hrp/adaag014

**Published:** 2026-09-05

**Authors:** Simrat Kaur Veera, Nitsan Shacham, Jessy Epstein, Danielle Wolfe Cohen, Elizabeth Roman, Kristen M. Thomas, Marissa Lipton, Brenda Kohn

**Affiliations:** a Division of Pediatric Endocrinology, NYU Grossman School of Medicine, New York, NY, USA; b Division of Pediatric Hematology/Oncology, NYU Grossman School of Medicine, New York, NY, USA; c Division of Pathology, NYU Grossman School of Medicine, New York, NY, USA; d Division of Pediatric Nephrology, NYU Grossman School of Medicine, New York, NY, USA

**Keywords:** Adrenocortical tumor, Adrenocortical adenoma, Cushing’s syndrome, Hypercortisolism, Hyperestrogenism

## Abstract

**Introduction:**

Adrenocortical tumors (ACTs) are rare pediatric tumors which include both benign adrenocortical adenomas (ACAs) and malignant adrenocortical carcinomas (ACCs). The most common presentations of an ACT in children are virilization and/or Cushing’s syndrome resulting from tumoral secretion of androgens and cortisol, respectively. Adrenocortical tumors that express high levels of estrogen are extremely rare, and adrenal tumors that co-secrete more than one class of adrenal hormones raise concern for malignancy.

**Case Presentation:**

We present a case of a 17-year-old female presenting with weight gain, secondary amenorrhea, and acute abdominal pain. She was found to have a large (9.4 cm) right adrenal tumor co-secreting both cortisol and estrogen, which was successfully surgically removed by minimally invasive laparoscopy. Her perioperative course was complicated by severe refractory hypertension requiring multiple antihypertensive medications, and venous thromboses associated with hypercoagulability. Genetic testing revealed no germline driver mutation. Classification of the adrenal tumor as either benign or malignant was discordant when determined by the adult Weiss vs. the pediatric AFIP/Wieneke criteria in this older adolescent.

**Conclusion:**

This prismatic case of a rare estrogen-cortisol secreting tumor raises discussion regarding the complexities in the clinical presentation and uncertainties in prognosis of adrenal tumors in adolescents. A literature review showcases 19 case reports published between 1948 and 2025 of estrogen-secreting pediatric ACTs highlighting their rare prevalence. This case stresses the need for a multidisciplinary approach to diagnosis, treatment, and long-term management of this entity in children and adolescents.

**Established Facts**

- Adrenocortical tumors are rare in pediatric and adolescent populations.
- Hormone secretion patterns in pediatric adrenocortical tumors are typically associated with hyperandrogenism and/or hypercortisolism. Estrogen-secreting tumors are rare.
- The AFIP/Wieneke criteria is the preferred risk stratification score in children with adrenocortical tumors; the Weiss criteria is standard in the adult population.

**Novel Insights**

- Variable clinical presentation of rare adrenocortical tumors can lead to misdiagnosis and delay in recognition.
- This case highlights the uncertainties in risk stratification using existing criteria in the older adolescent with adrenocortical tumors.
- A multidisciplinary, individualized approach is warranted for optimal diagnostic, therapeutic, and follow-up care of children and adolescents with adrenocortical tumors.

## Introduction

Adrenocortical tumors (ACTs) are uncommon in pediatrics and present a diagnostic and therapeutic challenge. Most ACTs in children are hormone-secreting, and present with clinical signs and symptoms of increased hormone production [1–5]. Cushing’s syndrome due to excess cortisol production, and isosexual or contrasexual precocity due to tumoral androgen production are among the most commonly reported presentations [1, 2, 6]. Estrogen-secreting adrenal tumors and ACTs that co-secrete cortisol and estrogen are exceedingly rare [6–9]. We report an older adolescent female who presented with severe Cushing’s syndrome and hyperestrogenism due to a large (9.4 cm) right adrenal mass. Biochemical evaluation demonstrated marked elevations in cortisol, estradiol, and estrone. The tumor size, age at presentation, and co-secretion of multiple hormones raised concern for malignancy. The patient underwent an adrenalectomy, and the tumor was classified as an adenoma based on the pediatric Armed Forces Institute of Pathology (AFIP)/Wieneke criteria, but demonstrated features concerning for malignancy by the adult Weiss criteria. This case highlights the complexities in the diagnosis and management of ACTs in adolescent patients.

## Case Presentation

### Patient Information

This case report was written in accordance with the CARE guidelines (see online suppl. material; for all online suppl. material, see https://doi.org/10.1159/hrp/adaag014). A 17-year-old Antiguan female presented with a 2-week history of sharp right upper quadrant abdominal pain radiating to the back. She reported a 90 kg (200 lb) weight gain over the prior 2 years, associated with the appearance of diffuse striae and secondary amenorrhea. Menarche had occurred at age 12 years, with regular menses until age 15 years, at which time menses ceased. The patient sought medical attention in Antigua and received a diagnosis of polyendocrine metabolic ovarian syndrome (PMOS). The possibility of Cushing’s syndrome was not considered for evaluation at that time. The patient was not on any medications. Family history was unremarkable.

### Clinical Findings

On admission, weight was 169.8 kg and height was 167.5 cm with a body mass index (BMI) of 59.1 kg/m^2^. Blood pressure was elevated at 180/110 mm Hg. On physical examination, the patient had Cushingoid features including moon facies, a dorsocervical fat pad, exophthalmos, and violaceous striae on the trunk and extremities (shown in Fig. 1a). She was Tanner stage V for both breast and pubic hair development. There was no hirsutism, acne, or clitoromegaly.

**Fig. 1.**
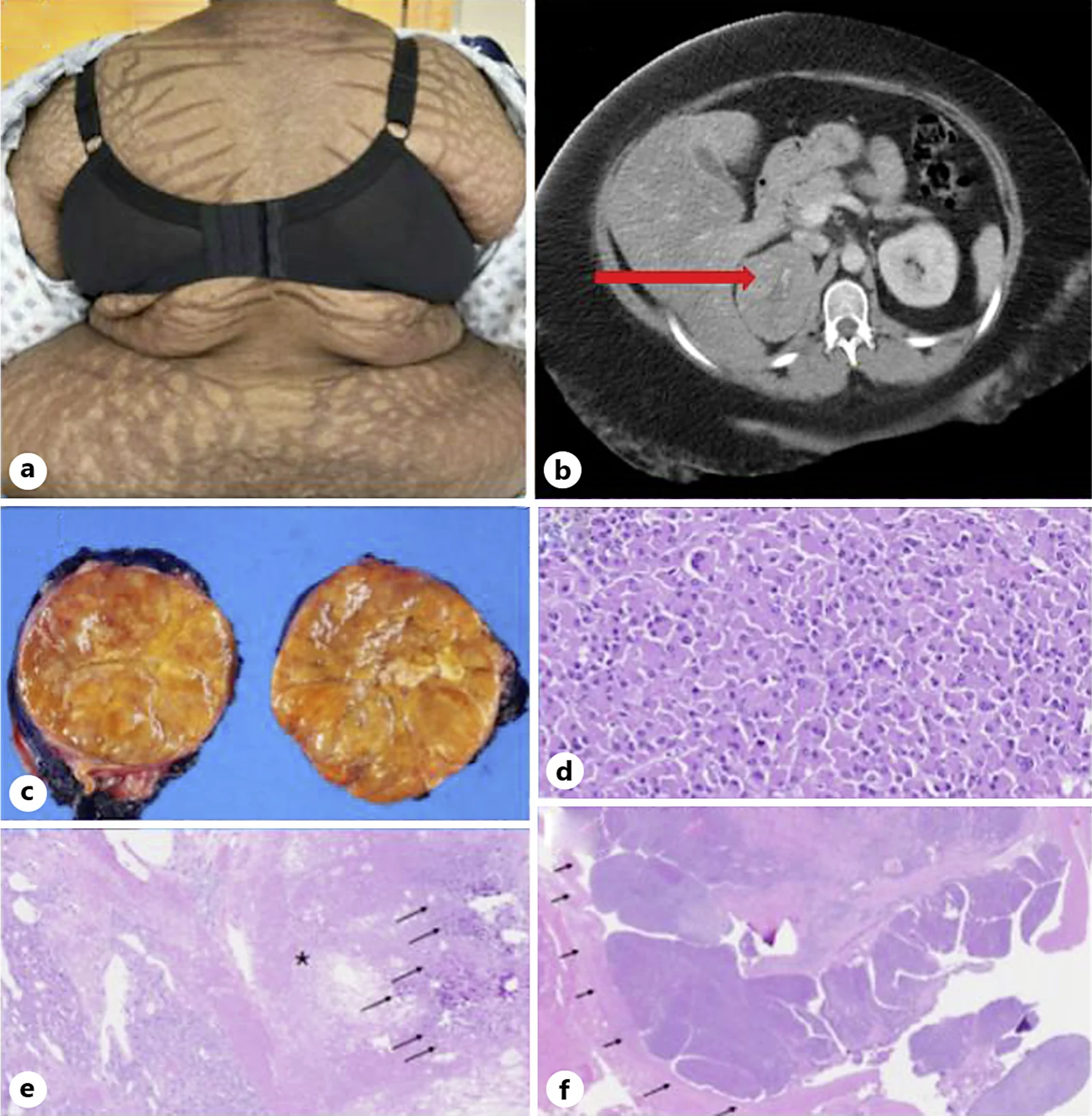
**a** Marked cushingoid appearance with deep, violaceous striae. **b** Axial contrast enhanced CT highlighting the right adrenal mass (arrow). **c** Gross specimen of the adrenal mass, 9.4 × 5.8 × 4.7 cm, weighing 147.9 g. Vaguely nodular, lobulated, golden to orange-yellow with central area of necrosis and calcification (paler area). **d** Oncocytic tumor cell morphology depicted with large, polygonal shaped cells with abundant eosinophilic cytoplasm and large, round nucleoli. There is limited pleomorphism and no evidence of mitotic figures. **e** Necrosis (*) and dystrophic calcification (arrows). **f** Adrenal mass within the adrenal vein (arrows pointing to edge of vessel wall).

### Diagnostic Assessment and Therapeutic Intervention

Abdominal ultrasound revealed a large right suprarenal mass and hepatic steatosis. CT and MRI of the abdomen confirmed a 7.4 × 5.9 × 4.8 cm right adrenal mass with central calcification (shown in Fig. 1b). Endocrine workup (Table 1) included an elevated serum AM cortisol at 47.1 μg/dL with a simultaneous undetectable ACTH, elevated serum estradiol 2,880 pg/mL (reference <649 pg/mL), elevated estrone >10,000 pg/mL (reference 4–10 pg/mL), elevated testosterone 141 ng/dL (reference <62 ng/dL), elevated sex-hormone binding globulin (SHBG) 342 nmol/L (reference 19–145 nmol/L), and undetectable gonadotropins. Serum 11-deoxycortisol was elevated at 308 ng/dL (reference <47 ng/dL), deoxycorticosterone (DOC) was undetectable <16 ng/dL (reference <19 ng/dL), while progesterone, 17-hydroxyprogesterone, pregnenolone, 17-hydroxypregnenolone, corticosterone, 18-hydroxycorticosterone, dehydroepiandrosterone sulfate (DHEAS), dehydroepiandrosterone (DHEA), and androstenedione were within normal ranges. Hypercortisolism was confirmed by an elevated urinary cortisol-to-creatinine ratio of 512 μg/g (ref <25 μg/g) and lack of suppressed morning serum cortisol 44.5 μg/dL (reference <1.8 μg/dL) following a modified low-dose (2 mg) oral dexamethasone suppression test taking the patient’s elevated BMI into account [10]. Evaluation for pheochromocytoma was negative and included urine vanillylmandelic acid (VMA) and homovanillic acid (HVA), as well as plasma and urine catecholamines, metanephrines, and normetanephrines. Chromogranin A was within normal limits at 43 ng/mL (reference <187 ng/mL). Thyroid function tests and hemoglobin A1c were within normal range. Fasting lipid profile showed a mildly elevated triglyceride and low-density lipoprotein level. Evaluation for metastatic disease, including chest CT, nuclear medicine bone scan, and PET-CT, was negative.

**Table 1.** Serum and urine evaluations

Laboratory evaluation	Pre-op	10 days post-op	15 days post-op
*Adrenal steroid work-up*			
AM ACTH (7.2–63.3 pg/mL)	<1.5	​	​
AM cortisol (<19.4 μg/dL)	47.14	​	​
Pregnenolone (26–235 ng/dL)	49	<5	​
Progesterone (<0.2 ng/mL)	0.4	​	​
Deoxycorticosterone (DOC) (<19 ng/dL)	<16	​	​
Corticosterone (130–820 ng/mL)	326	​	​
18-OH corticosterone (9–58 ng/dL)	8	​	​
Aldosterone (<31 ng/dL)	11.1	​	​
Renin plasma activity (0.5–4.0 ng/mL/h if upright, 0.2–1.6 ng/mL/h if supine)	13	​	​
17-OH pregnenolone (<412 ng/dL)	395	<25	​
17-OH progesterone (<179 ng/dL ng/dL)	64.4	<5	​
11-deoxycortisol (<47 ng/dL)	308	<10	<10
AM cortisol after 2 mg midnight dexamethasone suppression test (<1.8 mg/dL)	44.5	​	​
Cortisol in 24h urine (<56 μg/d)	810.9	​	​
Cortisol/Cr ratio in 24h urine (<24 μg/g Cr)	512.9	​	​
Cortisol, saliva (<0.1 μg/dL)	1.264	​	​
DHEA-S (63–373 μg/dL)	90	​	3
DHEA (1.24–7.88 ng/mL)	3.97	​	​
Androstenedione (0.35–2.12 ng/mL)	1.711	​	0.032
Testosterone (<6 ng/mL)	103	​	<1
Estrone (4–10 pg/mL)	>10,000	​	​
Estradiol (<649 pg/mL)	1,735	<10	3.4
*Adrenal medullary/pheochromocytoma work-up*			​
Urine metanephrines, 24h Ur/Cr ratio (0–320 μg/g Cr)	35	​	​
Urine normetanephrine, 24h Ur/Cr ratio (0–450 μg/g Cr)	200	​	​
Urine catecholamines, 24h Ur/Cr ratio	​	​	​
Dopamine (120–450 μg/g Cr)	247		
Norepinephrine (4–102 μg/g Cr)	37		
Epinephrine (3–58 μg/g Cr)	2		
Urine VMA Ur/Cr ratio (0–9 mg/g Cr)	2	​	​
Urine HVA Ur/Cr ratio (0–15 mg/g Cr)	2	​	​
Chromogranin A (0–187 ng/mL)	43	​	​
Serum metanephrines (0–0.49 nmol/L)	<0.10	​	​
Serum normetanephrines (0–0.89 nmol/L)	0.39	​	​
Serum catecholamines:	​	​	​
Dopamine (<240 pmol/L)	221		
Epinephrine (<330 pmol/L)	<55		
Norepinephrine (<3,100 pmol/L)	2,318		
*Additional endocrine markers*			
IGF-1 (120–479 ng/mL)	69	​	​
IGF-2 (127–473 ng/mL)	397	​	​
Prolactin (4–26 ng/mL)	41.4	​	​
*Metabolic labs*			
Glucose (70–99 mg/dL)	136	85	​
Sodium (136–146 mmol/L)	145	140	​
Potassium (3.5–5.0 mmol/L)	4.2	3.1	​
Chloride (98–106 mmol/L)	100	97	​
CO2 (24–31 mmol/L)	30	30	​
BUN (5.0–18.0 mg/dL)	18.0	15	​
Creatinine (0.5–0.9 mg/dL)	0.88	0.67	​
Calcium (8.4–10.2 mg/dL)	8.7	8.4	​
AST (10–35 U/L)	31	76	​
ALT (0–31 U/L)	51	153	​
Albumin (2.8–5.7 g/dL)	3.7	3.4	​
Total cholesterol (<170 mg/dL)	198	​	​
Triglycerides (<150 mg/dL)	201	​	​
HDL cholesterol (>39 mg/dL)	46	​	​
LDL cholesterol, calculated (<100 mg/dL)	112	​	​
Lipoprotein (a) (<29 mg/dL)	20	​	​
Thyroid stimulating hormone (0.5–4.3 uIU/mL)	2.44	​	​
Hemoglobin A1c (<5.7%)	4.1	​	​

The patient briefly received intravenous (IV) labetalol 10 mg upon initial evaluation in the emergency room due to her severe hypertension. This was discontinued once the adrenal mass was confirmed, and calcium channel blockers were instituted: a continuous nicardipine drip (maximum 15 mg/h) to allow for slow titration of her blood pressures prior to starting oral nifedipine (maximum 90 mg daily). Diuretics including oral spironolactone (maximum 100 mg every 12 h) and IV furosemide (maximum 40 mg daily) were added to address peripheral edema. The patient also received prophylactic treatment for hypercoagulability with low molecular weight heparin (LMWH) 40 mg subcutaneously every 12 h.

A right adrenalectomy was performed via minimally invasive laparoscopy. Stress-dose hydrocortisone was initiated with a loading dose of IV hydrocortisone 50 mg/m^2^ at the time of right adrenal vein clamping followed by IV hydrocortisone 100 mg/m^2^/d divided every 4 h for the first 24 h peri-operatively, to prevent an adrenal crisis. The resected mass was 9.4 cm in greatest dimension and 147.9 grams. Gross pathology revealed a multinodular, lobulated, well-circumscribed tumor with gross necrosis and central calcification. Microscopic examination (shown in Fig. 1d–f confirmed a well-circumscribed ACT composed predominantly of polygonal cortical cells admixed with small vessels and capillary-like structures. The cortical cells had a variable histological appearance, including a prominent oncocytic phenotype characterized by abundant eosinophilic cytoplasm and large, moderately pleomorphic nuclei. Cortical cells with lipid rich and clear cytoplasm were also identified with relatively smaller nuclei and prominent nucleoli. There were scattered mitotic figures (3–5 per 20 high-power fields [HPF]) and the overall Ki-67 proliferation index (PI) was low (<10%). There were no atypical mitotic figures. Scattered foci of necrosis were noted throughout the tumor. The tumor extended to the venous vascular margin. There was no evidence of capsular invasion, extra-adrenal extension, or involvement of the vena cava. Immunohistochemical staining for p53 demonstrated a wild-type phenotype, characterized by a heterogeneous staining pattern.

Genetic testing failed to identify a driver mutation in the tumor or germline mutation associated with inherited cancer predisposition syndromes. Next-generation sequencing performed on tumor DNA identified 2 variants of uncertain significance: a frameshift variant in the *MAP2K2* gene (c.1167_1168insGTTCAGCCGCAGGGTTTTACAC; p.Asn390ValfsTer30), and a frameshift variant in the *MTOR* gene (c3978del; p.Trp1326CysfsTer4). Focal gains on chromosome 16 and segmental losses involving chromosomes 1, 3, 4, 9, 10, and 11 were also noted (Genome PACT Program, NYU Langone). RNA sequencing of the tumor did not identify any gene fusions (FUSION-SEQer, NYU Langone). Circulating serum RNA analysis was negative for alterations in 76 genes associated with inherited cancer predisposition conditions, including *TP53* (CancerNext expanded + RNAInsight panel, Ambry Genetics). Whole-exome sequencing and whole-genome sequencing were not pursued. Based on these gross and histological features in the context of the pediatric AFIP/Wieneke criteria and taking the genetic and molecular findings into account for prognosis, the tumor was classified as a benign ACA (Table 2).

**Table 2.** Comparison of AFIP/Wieneke vs. Weiss scoring for our patient

Wieneke criteria	Case presentation	​	Weiss criteria	Case presentation
Tumor weight >400 g	✖		High nuclear grade	✔
Tumor size >10.5 cm	✖		Eosinophilic tumor cell cytoplasm (>75%)	✔
Extension into periadrenal soft tissues and/or adjacent organs	✖		Diffuse architecture (>33%)	✔
Invasion into vena cava	✖		Sinusoidal invasion	✖
Venous invasion	✔		Venous Invasion	✔
Capsular invasion	✖		Capsular Invasion	✖
Presence of tumor necrosis	✔		Necrosis	✔
Mitotic rate >15/20 HPF	✖		Mitotic rate > 5/50 HPF	✖
Atypical mitotic figures	✖		Atypical mitotic figures	✖
**Total score out of 9**	**2**		**Total score out of 9**	**5**
*Classification based on criteria met*			*Classification based on criteria met*	
0–2 = benign			0–2 = benign	
3 = intermediate for malignancy			3+ = malignant	
4+ = malignant				

### Follow-up and Outcomes

Serum estrogens measured low on evaluation 10 days after surgery (Table 1) confirming the tumor as the source of excess hormone production. Cortisol was not measured as the patient continued on a slow post-operative steroid taper.

The hospital course was further complicated by an upper extremity superficial venous thrombosis and a lower extremity deep vein thrombosis (DVT), for which the patient received therapeutic anticoagulant treatment with oral apixaban 5 mg every 12 h for 6 weeks with complete resolution of the DVT. Hypertension was managed post-operatively with calcium-channel blockers and diuretics. Enalapril was also added for the severity of the hypertension. Hypertension gradually improved and the patient was able to discontinue all anti-hypertensives by 7 weeks post-surgery. The patient also had an episode of breakthrough menstrual bleeding 3 weeks post-operatively and has since required medroxyprogesterone acetate, a progestin, to reinstate menstrual cycles, which continue to be irregular.

Surveillance over the next 4 months post-operatively included a repeat MRI of the abdomen and pelvis with no recurrent disease and a DEXA scan which showed low bone density of the spine (Z score −3.3). The patient was also noted to have a 2.7 kg (5 lb 15 oz) decrease in weight during this time period.

The patient has since returned to Antigua. She is being monitored by a group of subspecialists. She continues on a surveillance protocol with clinical, hormonal, and imaging evaluations including adrenal MRI every 4–6 months [2]. She is currently maintained on physiologic hydrocortisone replacement and will continue to be tapered over the next year with anticipated recovery of the hypothalamic-pituitary-adrenal axis.

## Discussion

ACTs are rare pediatric tumors comprising both benign adrenocortical adenomas and malignant adrenocortical carcinomas. The reported incidence of pediatric ACTs ranges from 0.1 to 1.67 cases per million, with a significantly higher incidence of approximately 3.4 per million in Southern Brazil, attributed to a germline pathogenic p.R337H variant in *TP53* [1, 3, 11–15]. A bimodal distribution has been described, with peaks occurring between 1 and 4 years of age and again during adolescence. The early peak may reflect tumorigenesis arising from an impact on the embryonal adrenal cortex whereas the later peak may be associated with changes during adrenarche [1, 3, 5, 16, 17]. In contrast to adult ACTs, pediatric ACTs are more frequently hormone-secreting [1, 5, 18]. The most common clinical presentations in children include Cushing’s syndrome resulting from cortisol excess and precocious puberty with virilization resulting from androgen excess [1, 18]. Hyperestrogenism, hyperaldosteronism, and co-secretion of multiple hormones are rarely reported [6–9].

Advances in molecular genetics have furthered our understanding of genetic and epigenetic drivers of pediatric ACTs. Pediatric ACTs are associated with cancer predisposition syndromes including Li-Fraumeni syndrome, Beckwith-Wiedemann syndrome (BWS), and multiple endocrine neoplasia type 1 [3, 17–19]. *TP53* mutations, such as those associated with Li-Fraumeni syndrome, are strongly implicated in the pathogenesis of pediatric ACTs [3, 19]. However, approximately 50% of pediatric ACTs arise in the absence of germline *TP53* mutations (*TP53* wild-type) [3, 12]. In these cases, alternative oncogenic drivers, including activating mutations in *CTNNB1*, have been identified, although the molecular mechanisms underlying tumorigenesis in *TP53* wild-type ACTs remain incompletely understood [3]. ACTs associated with chromosome 11p15 abnormalities, as seen in BWS, have been associated with both wild-type or mutated *TP53* cases and may overexpress IGF-2 [3, 18].

Determination of the malignant potential of ACTs has historically relied on criteria derived from adult data and differentiating between benign and malignant pediatric ACTs has been challenging [14, 20]. The Weiss scoring system, first proposed in 1984, is based on nine histopathological parameters used to distinguish benign ACAs from malignant ACCs (Table 2). These include high nuclear grade, mitotic rate >5 per 50 HPF, atypical mitotic figures, <25% clear cells, >33% diffuse architecture, necrosis, venous invasion, sinusoidal invasion, and capsular invasion [21, 22]. In 2002, a modified Weiss system was proposed to reduce subjectivity and sampling bias, focusing on five weighted features: mitotic rate >5 per 50 HPF, <25% clear cells, atypical mitoses, necrosis, and capsular invasion [23]. However, the Weiss-based criteria has been shown to overestimate malignant potential in pediatric ACTs, leading to overtreatment with chemotherapy [20, 24]. In children, features such as eosinophilic cytoplasm, diffuse architecture, and sinusoidal invasion have less prognostic significance compared to adults, whereas clinical features including tumor weight and size are more predictive of outcome [20]. The AFIP/Wieneke scoring system, proposed in 2003, is now the standard for pediatric ACT classification and incorporates nine parameters: tumor weight >400 g, tumor size >10.5 cm, extension into periadrenal tissue or adjacent organs, invasion into the vena cava, venous invasion, capsular invasion, necrosis, mitotic rate >15 per 20 HPF, and atypical mitotic figures (Table 2) [20]. This system has repeatedly demonstrated an improved prognostic predictive index (PI) in clinical outcomes for younger children [25–27]. Though tumor volume and weight have been studied to significantly correlate with clinical outcomes in pediatric ACTs, it should be noted that the size and weight cutoffs in the Wieneke criteria were defined from a series of relatively advanced cases, so there is a possibility that further studies looking at lower cutoffs may provide stronger prognostic utility [28]. Additionally, a Ki-67 PI ≥15% and older age > 10 years old at presentation have been independently associated with poorer outcomes in pediatric ACTs [3, 24]. In cases of advanced disease, aberrant growth, and high Ki-67 PI, treatment may include adjuvant mitotane and cisplatin-based chemotherapy in addition to surgical resection [2, 5, 29].

Table 3 summarizes 19 reported cases of estrogen-secreting adrenal tumors in patients younger than 24 years of age published between years 1948 and 2025 [6, 30–46]. Notably, a subset of these cases were reported prior to the adoption of the Weiss or the AFIP/Wieneke criteria. As such, classification of those tumors may differ when re-evaluated using present standards.

**Table 3.** Literature review of published cases of estrogen-secreting adrenal tumors in children and young adults

Reference [#]	Age, years	Sex	Clinical presentation	Hormone secretions	Tumor classification (and criteria if mentioned)
Arnautu et al. [30] (2025)	24	Male	Right upper quadrant pain, bilateral gynecomastia, infertility	Elevated estradiol	Adrenocortical carcinoma per Weiss criteria
Verma et al. [31] (2022)	4	Female	Early breast development, menstrual bleeding, pubic hair, and clitoromegaly	Elevated estradiol and DHEAS	Adrenocortical oncocytoma (benign) per modified Weiss criteria
Tseretopoulou et al. [32] (2022)	7	Female	Early breast development, vaginal discharge, pubic and axillary hair	Elevated estradiol, and a LH-releasing hormone test produced elevated adrenal androgens	Adrenal adenoma per Wieneke criteria
Vuralli et al. [6] (2022)	13.5	Male	Bilateral gynecomastia and cushingoid features	Elevated estradiol, androgen levels, cortisol, 17OHP, and 11DOC	Adrenocortical carcinoma with pulmonary metastasis
Vuralli et al. [6] (2022)	7	Female	Early breast development and pubic hair	Elevated estradiol, testosterone, and DHEAS	Unspecified ACT
Elnaw et al. [33] (2021)	4	Female	Early breast development, growth acceleration	Elevated estradiol, DHEAS	Adrenocortical adenoma per Wieneke criteria
Bouyahia et al. [34] (2009)	5	Female	Early breast development, pubic hair, menses	Elevated estradiol, testosterone, DOC	Adrenocortical adenoma
Shen et al. [35] (2009)	19	Male	Presentation not specified	Elevated estrogen	Adrenocortical carcinoma per Weiss criteria
Ghazi et al. [36] (2004)	5.5	Female	Rapid linear growth, clitoromegaly, acne, temporal hair loss, and pubic hair	Elevated testosterone, cortisol, estradiol	Adrenocortical adenoma
Watanabe et al. [37] (2000)	1.5	Male	Bilateral gynecomastia, facial acne, and pubic hair	Elevated estradiol, testosterone, DHEAS, increased aromatase activity on tumoral tissue sampling	Adrenal adenocarcinoma
Balakumar et al. [38] (1997)	6	Male	Bilateral gynecomastia, pubic hair, acne, body odor, and facial plethora	Elevated estradiol, testosterone, DHEAS, androstenedione, and cortisol	Adrenocortical adenoma
Kimura et al. [39] (1995)	19	Male	Gynecomastia, marked obesity, acne on back, and hypertension	Elevated estradiol, estrone, 11-DOC, DOC, mildly elevated cortisol. Elevated aromatase activity on tumoral tissue sampling	Adrenocortical carcinoma
Ghazi et al. [40] (1994)	14	Male	Bilateral gynecomastia, weight gain, Cushingoid features, acute onset of aggressive behavior and delirium	Elevated estradiol, cortisol, testosterone, prolactin	Unspecified adrenal tumor
Itami et al. [41] (1982)	6	Male	Bilateral gynecomastia, growth acceleration, light facial hair, pubic hair	Testosterone, estradiol, estrone, 11-DOC, 17-OHP, DHEAS, androstenedione	Adrenal adenoma
Drop et al. [42] (1981)	6.5	Female	Growth acceleration, early breast development, pubic hair, menses	Elevated estradiol, prolactin, 11-DOC, DOC, DHEAS, DHEA, androstenedione, urinary 17-OH steroids	Adrenal carcinoma
Schteingart et al. [43] (1979)	22	Female	Secondary amenorrhea, hirsutism, acne, fatigue, slight weight gain, mild clitoromegaly	Testosterone, cortisol, DHEAS, estradiol, slightly elevated urinary 17-OH steroids	Adrenocortical adenoma
Sultan et al. [44] (1979)	15	Male	Bilateral gynecomastia	Elevated serum and urinary estrogen, urinary 17 ketosteroids	Adrenal adenoma
Bhettay et al. [45] (1977)	6	Male	Bilateral gynecomastia	Elevated urinary estrogens	Adrenocortical adenoma
Wilkins [46] (1948)	4.75	Male	Bilateral gynecomastia since infancy	Elevated urinary estrogens	Adrenal adenoma

Our case highlights the presentation of an ACT in an older adolescent with elevated levels of estrone and estradiol in co-secretion with cortisol, manifesting as secondary amenorrhea and Cushing’s syndrome. The tumor was initially suspected to be malignant given the patient’s age, large tumor size, presence of calcification on imaging, and rare combination of estrogen and cortisol co-secretion. Histologically, the tumor met 5 of 9 Weiss criteria (high nuclear grade, <25% clear cells, >33% diffuse architecture, venous invasion, and necrosis) which would support the diagnosis of malignancy in adults. In contrast, based on the pediatric AFIP/Wieneke criteria, the tumor met only 2 parameters for malignancy (venous invasion and necrosis) (Table 2). Extensive genetic testing excluded a cancer predisposition syndrome and demonstrated a wild-type *TP53* phenotype with a low Ki-67 PI, further indicative of a favorable prognosis.

The combined impact of hypercortisolism and hyperestrogenism created significant clinical challenges, requiring safe management of severe hypertension and hypercoagulability. Labetalol, a β-blocker antihypertensive, was discontinued and calcium channel blockers were initiated upon admission. Calcium channel blockers were selected based on their paucity of adverse effects, rapid onset of action, and non-inferiority to α*-*blockers in pheochromocytomas [47, 48]. Pheochromocytoma was carefully excluded (Table 1). The patient’s hypertension was attributed to the severe hypercortisolism overwhelming the capacity of 11β-hydroxysteroid dehydrogenase type 2, an enzyme that normally protects the mineralocorticoid receptor by converting cortisol to inactive cortisone [49]. 11-deoxycortisol was elevated, attributed to disorganized steroidogenesis or “backflow” from the excess cortisol production [50]. As a weak mineralocorticoid, it may have also contributed to the severity of the hypertension. Enalapril, an angiotensin-converting enzyme inhibitor, was added to the antihypertensive regimen to target activation of the renin-angiotensin-aldosterone system (RAAS), which may have been driven by hypercortisolism-induced angiotensinogen production. The patient’s mildly elevated plasma renin activity (Table 1) may also reflect enhanced RAAS activation secondary to hyperestrogenism, as estrogen stimulates hepatic angiotensinogen synthesis, potentially further contributing to the development of hypertension [51, 52].

Estrogen hypersecretion in ACTs has been described in the literature and may be mediated by increased aromatase (*CYP19A1*) activity in the tumor, resulting in conversion of androgen precursors into estrogens [53]. Furthermore, 17β-hydroxysteroid dehydrogenase enzymes catalyze the interconversion between estradiol and estrone and may exhibit altered activity in ACTs, contributing to elevations in both hormones [54]. The hyperestrogenism, in combination with the patient’s extremely elevated BMI and post-surgical immobility, likely also significantly increased the patient’s risk of hypercoagulability [55].

This case highlights the rarity of estrogen and cortisol co-secreting ACTs in adolescents and underscores the potential for delayed diagnosis when clinical manifestations are initially attributed to more common conditions such as PMOS. It also illustrates the diagnostic and prognostic challenges of applying existing risk stratification systems to older adolescents, in whom the distinction between pediatric and adult disease paradigms becomes increasingly indistinct as physiologic maturation approaches adulthood. In our patient, application of adult Weiss criteria suggested malignant potential, whereas pediatric AFIP/Wieneke criteria, together with favorable molecular features, supported a more benign classification. This discordance emphasizes the limitations of current prognostic frameworks in this transitional age group and highlights the need for continued refinement of risk stratification models for pediatric ACTs.

Although we continue to support the AFIP/Wieneke criteria as the gold standard for pediatric ACTs, cautious long-term surveillance remains warranted even in cases with otherwise favorable pathologic features. Management of pediatric ACTs should be multidisciplinary, extending beyond tumor-directed therapy to address the full spectrum of endocrine, metabolic, and systemic sequelae. Given the limited published data on long-term outcomes in older adolescents and based on our patient’s individualized factors, we recommend that serial hormonal evaluations and adrenal MRIs every 4–6 months during the initial post-resection years are essential to facilitate early detection of recurrence or delayed malignant behavior [2]. Finally, well-designed prospective multicenter studies with long-term follow-up are needed to better define prognostic factors, validate existing pediatric risk stratification systems in older adolescents, and establish evidence-based surveillance guidelines for this rare patient population.

## Acknowledgment

We thank Dr. Justin Loona for providing radiological imaging.

## Statement of Ethics

Ethical approval is not required for this study in accordance with local or national guidelines. Written informed consent was obtained from the patient and her guardian for publication of the details of her medical case and photo images.

## Conflict of Interest Statement

The authors have no conflicts of interest to declare.

## Funding Sources

We acknowledge the support of the Dr. Raphael and Dr. Irene David Pediatric Endocrinology Research Fund at NYU Langone Health, NYU Grossman School of Medicine. The funder had no role in the design, data collection, data analysis, and reporting of this study.

## Author Contributions

S.K.V., N.S., J.E., and D.C. led the analysis and writing of the paper. K.M.T. provided the histological images and descriptions. K.M.T., E.R., M.L., and B.K. oversaw the progress and provided critical revisions. All contributing authors approved the final version of this manuscript.

## Data Availability

All data generated or analyzed during this study are included in this article. Further enquiries can be directed to the corresponding author.
